# The impact of maternal influences on childhood obesity

**DOI:** 10.1038/s41598-022-10216-w

**Published:** 2022-04-15

**Authors:** Pei-Chuan Hsu, Fang-Ming Hwang, Mei-I Chien, Wui-Chiu Mui, Jyh-Mirn Lai

**Affiliations:** 1 Department of Early Childhood Educare, Tainan University of Technology, Tainan City, 710 Taiwan; 2 Department of Education, National Chiayi University, Chiayi City, 600 Taiwan; 3 Department of Early Childhood Education, National Chiayi University, Chiayi City, 600 Taiwan; 4 Chiayi Christian Hospital, Chiayi City, 600 Taiwan; 5 College of Veterinary Medicine, Department of Veterinary Medicine, National Chiayi University, Chiayi City, 600 Taiwan

**Keywords:** Health care, Health services, Public health

## Abstract

There was a lack of detailed information about maternal influences on their children’s body mass index (BMI) in Taiwan. The aim of this study was to find the evidence to describe how mothers’ factors could affect their 2 to 9-year-old children’s BMI, with data collected from May 2021 to June 2021. Anonymous self-administered questionnaires were completed by 1035 participants from Taiwan’s six metropolitan cities and eight counties. After controlling for children’s factors, such as number of children in a family, children’s constitution, children’s age and gender, hierarchical regression models were used to analyze the effects of five maternal factors on their children’s BMI: maternal BMI, age, education level, monthly household income, and marital status (single parent or not). The results were found as follow: maternal BMI [*β* = .24], maternal educational level [*β* = −.141], and monthly household income [*β* = .071], significantly (*p* < 0.05) influenced their children’s BMI. Higher maternal BMI was associated with a higher children’s BMI. Mothers with lower levels of education background were more likely to have children with a higher BMI. Monthly household income was a positive factor influencing children’s BMI. In conclusion, this study is the first detailed description of maternal influences on their 2–9 years old children’s BMI in Taiwan. Although the study could not cover all of the factors influencing Taiwan’s childhood obesity, we have discovered maternal BMI, education level, and monthly household income were significant factors associated with children’s BMI.

## Introduction

Childhood obesity has become a big public health challenge worldwide. In Taiwan, the percentage of overweight school children increased from 19.8 to 26.1% between 1993 and 2018, which meant one in four children was overweight or obese^1^. Childhood obesity is linked to many negative outcomes such as chronic diseases and adult obesity^2,3^, attention deficit/hyperactivity disorder^4^, low self-esteem^5^, victims of bullying^6^, and stigma associated with obesity^7^.

Childhood obesity is a complex and multifactorial outcome^8^ which can be attributed to factors such as genes^9^, socioeconomic status (SES)^10^, race^11^, ethnicity^11^, gender^11^, lifestyle, and diet^12^. Some maternal factors, such as mother’s age and education level, and family income have been proven but their influences are still worth discussion. Younger mothers were more likely to have their 10–12 years old children with obesity^13^. However, mother over 40 years old were more like to raise obese seven-year-old children^14^. The findings from studies focusing on how family income influenced the children were also mixed. Parental income was a significant family-related factor associated with obesity among 6-year-old children in Sydney^15^ but not significant among children aged 0.1–6.9 in China^16^. Obesity was found to be related to high income in Sub-Saharan African countries but associated with low income in the USA^17^. Some believed that children in low-income families ate less healthy food^15^ or more junk food^18^. No difference by race was found in this analysis^18^. In regard to marital status, children were more likely to develop obesity if they lived with a signal parent or when they were the only child at home^19,20^ due to reasons such as limited resources, lack of exercise—especially in girls, unsecured living area, and lack of time to make a healthy meal. However, it was also found that when children became older and more independent, these factors were not statistically significant^19,21^. Previous studies on the relationship between maternal education and obese children have produced inconsistent results. Some studies have pointed out that a higher maternal education is associated with obese children^17^, while another report suggested university-educated parents are a protective factor of obesity in 6-year-old children^15^. However, some have also suggested that mothers with a higher level of education could provide a better dietary quality and nutritional food for their children and related to less overweight children^22^.

In Taiwan, previous studies mainly focused on the prevalence, causes and comorbidities caused by obesity^23–25^. The prevalence of obesity was 14.7% in boys and 9.1% in girls at Taiwan’s elementary schools in 2007^24^. High-calorie diets and sedentary behaviors were associated with obesity among first and second grade elementary students^26^. Obese or overweight children and adolescents aged 5 to 18 years old had a higher prevalence of comorbidities relative to those of a healthy weight group^26^. However, according to the authors’ knowledge, studies focusing on the factors related to obesity between maternal factors and their aged 2–9 years old children in Taiwan are rare. In addition, mothers are usually the primary caregivers in most societies^19^ and affect their children’s health attitudes, habits or behaviors, and their access to healthy food^26^. Thus, understanding the impact of maternal factors on childhood obesity may help Taiwan control obesity. The study sought to provide more information on the relationship between maternal factors and the obesity of Taiwan’s 2–9-year-old children.

## Results

Table 1 presents the distribution of demographic data of the children and their mothers. The majority of children were five years old (25.70%) with a mean age of 5.74 ± 1.50. The average age of mothers was 37.19 ± 4.67 years with a ranged from 23 to 55 years. 71% of the mothers were between 31 and 40 years old. Most respondents had a monthly household income between 50,000 and 80,000 New Taiwan Dollars (NTD), which were 316 (31.80%) of the samples. Only 14 (1.40%) of the families earned less than 15,000 $NTD which is similar to the data of the Ministry of Health and Welfare in 2021 (1.59%). The most common maternal status was children living with both parents (969 cases, 93.60%). Two-children families are the most common (649 or 62.70%). Of the 1035 surveyed mothers, 118 (11.4%) of their children were obese and 917 (88.60%) were not.

**Table 1:** Demographic characteristics of the children and mothers in the study.

Characteristics of sample (N=1035)	N (%)	Non-obese (%)	Obese (%)	*t* or *F* test	p_value
**Children’s gender**					
boys	500 (48.3%)	437 (42.4%)	63 (6.1%)	1.169	.242
girls	535 (51.7%)	48 (46.5%)	55 (5.3%)		
Children’s age	Mean$\pm \mathrm{SD}$ 5.74$\pm$1.50 y/o				
2 y/o	4 (0.4%)	4 (0.4%)	0 (0%)	.836	.557
3 y/o	63 (6.1%)	51 (4.9%)	12 (1.2%)	5>4>2 y/o	
4 y/o	154 (14.9%)	135 (13.0%)	19 (1.8%)		
5 y/o	266 (25.7%)	236 (22.8%)	30 (2.9%)		
6 y/o	229 (22.1%)	207 (20.0%)	22 (2.1%)		
7 y/o	174 (16.8%)	157 (15.2%)	17 (1.6%)		
8 y/o	111 (10.7%)	98 (9.5%)	13 (1.3%)		
9 y/o	34 (3.3%)	29 (2.8%)	5 (0.5%)		
Children’s constitution	Mean$\pm \mathrm{SD}$ 3.27$\pm$.69				
[1] very poor	7 (0.7%)	5 (0.5%)	2 (0.2%)	3.278^*^	.011
[2] poor	78 (7.6%)	71 (6.9%)	7 (0.7%)		
[3] fair	617 (59.9%)	560 (54.4%)	57 (5.5%)		
[4] Good	282 (27.4%)	241 (23.4%)	41 (4.0%)		
[5] very good	46(4.5%)	36(3.5%)	10(1.0%)		
Maternal age	Mean$\pm \mathrm{SD}$ 37.19$\pm$4.67 y/o				
[1] $\le$30	85 (8.3%)	70 (6.8%)	15 (1.5%)	1.223	.300
[2] 31-40	729 (71.0%)	649 (63.2%)	80 (7.8%)		
[3] 41-50	207 (20.2%)	185 (18.0%)	22 (2.1%)		
[4] $\ge$50	6 (0.6%)	5 (0.5%)	1 (0.1%)		
**Maternal education level**					
[1] Under junior high school	32 (3.2%)	28 (2.8%)	4 (0.4%)	4.512^**^	.001
[2] High/vocational school	215 (21.3%)	178 (17.7%)	37 (3.7%)	[2]>[4]>[5]	
[3] Technical college	153 (15.2%)	128 (12.7%)	25 (2.5%)		
[4] University	473 (46.9%)	429 (42.6%)	44 (4.4%)		
[5] Postgraduate	135 (13.4%)	128 (12.7%)	7 (0.7%)		
**Monthly household income (NTD)**					
[1] <15,000	14 (1.4%)	14 (1.4%)	0 (0.0%)	1.962	.068
[2] 15,000~30,000	81 (8.1%)	69 (6.9%)	12 (1.2%)		
[3] 30,000~50,000	224 (22.5%)	195 (19.6%)	29 (2.9%)		
[4] 50000~80000	316 (31.8%)	272 (27.4%)	44 (4.4%)		
[5] 80,000~100,000	163 (16.4%)	148 (14.9%)	15 (1.5%)		
[6] 100,000~150,000	144 (14.5%)	136 (13.7%)	8(0.8%)		
[7] >150,000	52 (5.2%)	48 (4.8%)	4 (0.4%)		
**Marital status (Single parent or not)**					
[1] Single parent	66 (6.4%)	56 (5.4%)	10 (1.0%)	.990	.322
[2] Parents	969 (93.6%)	861 (83.2%)	108 (10.4%)		
**The number of biological children in the family**					
[1] 1	190 (18.4%)	171 (16.5%)	19(1.8%)	.198	.898
[2] 2	649 (62.7%)	573 (55.4%)	76 (7.3%)		
[3] 3	167 (16.1%)	148 (14.3%)	19 (1.8%)		
[4] $\ge 4$	29 (2.8%)	25 (2.4%)	4 (0.4%)		

*p < 0.05; ** p < 0.01; *** p < 0.001.

The results from the hierarchical multiple regression models are shown in Table 2. The control variables were added in step 1. Results of the hierarchical multiple regression showed that children’s constitution, children’s age and children’s gender explained 3.9% of the variance in children’s BMI. There was no significant association between number of children in a family and children’s BMI. The maternal factors were included in step 2. In the second model, the R^2^ change was equal to 0.08 (F = 14.038, *p* < 0.001). The results showed that mother’s BMI (β = 0.240; *p* < 0.001), education level (β = −0.141; *p* < 0.001), and monthly household income (β = 0.071; *p* < 0.05) explained an additional 8% of the variance in children’s BMI and it was statistically significant. Maternal age and marital status, were non-significant factors.

**Table 2:** Hierarchical Liner Regression of Predictors of children’s BMI.

Predictor variables	Model 1	Model2
The number of biological children in the family	−0.036	−0.051
Children’s constitution	0.081^*^	0.084^**^
Children’s age	0.162^***^	0.152^***^
Children’s gender (1, boys; 2, girls)	−0.074^*^	−0.067^*^
Maternal BMI		0.240^***^
Maternal age		−0.065
Maternal education level		−0.141^***^
Monthly household income		0.071^*^
Marital status		−0.067
R^2^	0.039	0.119
F	9.612^***^	14.038^***^
△R^2^	0.035	0.110
R^2^ change	0.039	0.080

*p < 0.05;** *p* < 0.01;*** *p* < 0.001.

## Discussion

The study sought to understand whether maternal factors could predict the BMI of 2–9-year-old children in Taiwan. In our study, after controlling for children’s factors, we have successfully used five variables to predict the BMI in children. Similar to what other studies have suggested^27^, this study also found that variables such as maternal BMI, education level, monthly household income are the main factors affecting the children’s BMI^27^. BMI is a fair measurement to estimate whether people are overweight, but obesity is also influenced by many other factors, such as age, gender^28,29^ and could also be inherited^26^. In our model, maternal BMI (β = 0.24) and education level (β = −0.141) were the most important factors affecting the children’s BMI. It can also be explained by an additional change of R^2^ in our second model. This is similar to another study which suggested mother’s BMI could contribute to 25–40% of children’s BMI^30^. Higher educated mothers seem to have a more positive attitude toward healthy food.

One interesting result from our study was that a one-rank increase in the family income resulted in a 0.071 BMI increase in the regression model, a significant but rather mild contribution to the children’s BMI. A study, which reviewed papers from different countries (16 from the USA, three from the UK and two from Canada), found inconsistent results of the family income factor and assumed that it was due to the difficulty of getting negative results published because income had been well established in social epidemiological and public health researches as a risk to subsequent obesity^26^. We believe further research on the impact of family’s SES or income on children’s BMI may provide Taiwan a better profile about this issue.

Some researchers tend to use the term “parental perceptions of their child’s weight” to describe whether parents think their children are overweight^31,32^. Parents who have a higher “parental perceptions of their child’s weight (Likert-scale 1–3), are likely to have overweight children (Liker-scale 1–3). In our study, the variable “children’s constitution” was defined as children’s health condition perceived by their own mothers which admittedly is very subjective. A mother would give a higher score in “children’s constitution” if she thinks her child is healthy. In Taiwan, it is culturally normative to assume that a baby that has more body fat is a baby that is robust and healthy. We added this variable as a control to facilitate clearer information.

Some researchers believed that children without siblings were at a higher risk of becoming overweight and obese^21,33,34^. However, a recent study on seven reports found no association although the pooled OR was 1.46 and concluded that parents paid more attention in nutrition if they had less children^35^. Taiwanese families tend to have only one or two children and, in our study, 81.4% of participants had only one to two children. The Ministry of Education in Taiwan has been running campaigns for years such as Aid Students to Fit to control students’ weight and requires parents and teachers to work together in these campaigns. We believe this could be the reason why some variables were non-significant. Further research would be needed to determine the impact of sibling numbers on children’s BMI.

Our study found that maternal age and marital status did not significantly impact on the children’s BMI. We believe this is due to the strong family support in Taiwan and a low number of very young mothers. 70% of our respondents were mothers aged between 31 and 40 years old. Some researchers suggested that children with single parent might be likely to be obese^19,20^. The possible reasons could be a lack of resources, reduced physical activities, lacking time to prepare healthy foods, and uncertainties around housing^19,21^. Chen and Escarce^19^ suggested when children got older and more independent, this factor would become non-significant. We think there could be another reason in Taiwan. In our study, only 26 single mothers lived alone with their children, while the other 40 still lived with their original families and some even still lived with their ex-husbands. Hence, we believe a strong family support could overcome the negative outcomes of single parenthood.

## Limitations

This study has obtained a sample size (n = 1035) bigger than the minimum sample size calculated by G*power although, with a R square of 0.11, it may not be able to explain the full picture of the impact of maternal factors on children’s BMI.

This study is also limited in some other areas. Firstly, while many factors possibly affecting children’s BMI, this study only discussed maternal influences and did not consider influences from the fathers or other primary caregivers. Therefore, the results of this study cannot be inferred beyond the scope of the study. Secondly, the children’s constitution was perceived by their own mothers, which was not of any medical objective standard and very subjective. This is designed mainly to understand whether mother's subjective perception of their children's constitution would also contribute to their children’s BMI. However, more comprehensive related factors should be considered in future studies.

## Conclusion

In conclusion, as the first report in Taiwan to explore how maternal factors could impact on children’s BMI, this study proved that maternal BMI, education level, and monthly household income were significant factors affecting their children’s BMI. In the future, it may be possible to use structural equation modeling to analyze the association between parent’s factors and the obesity of Taiwan’s 2–9-year-old children.

## Methods

### Study design and samples

The survey was conducted between May and June 2021. 1,100 mothers who had at least one child aged between two and nine years old were invited to complete self-administered anonymous questionnaires. Data were collected through convenience sampling from 26 kindergartens and 20 elementary schools in Taiwan’s six metropolitan (New Taipei City, Taipei City, Taoyuan City, Taichung City, Tainan City, and Kaoshiung City) and 8 counties (Hsintsu city, Hsintsu county, Nantou county, Yulin County, Chiayi City, Chiayi County, Taitung County, Hualien County). Participants were informed about the purpose of the study and had given written consent. It is not possible to identify individual participants as all information were provided anonymously and indirectly to the researchers. The study was approved by National Cheng Kung University of IRB, which was the medical university that oversees research at our institution. The number was NCKU HREC-E-110-065-2. All methods were performed in accordance with the relevant guidelines and regulations. After deducting 65 responses which were deemed invalid due to a serious omission or failure to fill in the height and weight of the children, the remaining 1,035 valid questionnaires were used for this study. G*power 3.1.9.4, a tool to compute the sample size for multiple regression, was used to assess the appropriate sample size for this study.

### Variables

The independent variables were the maternal factors such as mother’s body mass index (BMI), age, education level, marital status (single parent or not), monthly household income, number of children in a family and children's constitution (Table 3). Monthly household income was defined as the total monthly income of the whole family. 5-point Likert scale was used to assess children’s constitution which referred to children’s health condition subjectively perceived by their own mother.

**Table 3:** Variable information.

Independent variables	
Maternal’ age	[1] <=30, [2] 31-40 y/o, [3] 41-50 y/o, [4]>=51 y/o
Maternal’ BMI	BMI was calculated by a formula which use a person’s weight(kg) divided by height (Meters, m^2^).
Maternal’ Education	[1] Under Junior high school, [2] Senior/vocational high school, [3] Junior/technique college, [4] University, [5] Postgraduate
Marital status	[1] Single parent, [2] Parents
Household income	[1] <15,000, [2] 15,000~30,000, [3] 30,000~50,000, [4] 50,000~80,000, [5] 80,000~100,000, [6] 100,000~150,000, [7] >150,000
The number of biological children in the family	[1] one, [2] two, [3] three, [4] four or above
Children’s gender	[1] boy, [2] girl
Children’s constitution	Mothers were asked to evaluate their children’s constitution according to the Likert 5-point scale classification method, which was divided into five levels: [1] stands for "very poor", [2] stands for "poor", [3] stands for "fare", [4] stands for "good" and [5] stands for "very good"
Outcome variable	
Children’s BMI	Children’s BMI was obtained according to the height and weight of the children measured by the school and converted the data to BMI [weight (Kg)/height (m^2^)].

The variate was the children’s body mass index (BMI). BMI was calculated as a person’s weight in kilograms divided by height in meters squared (kg/m^2^) published by the Health Promotion Administration, Ministry of Health and Welfare in Taiwan^36^.

### Anthropometry

Children’s height (cm) and weight (Kg) were measured during routine physical examinations by a health team from the region’s teaching hospitals at the start of each grade’s first semester. Results of the physical examinations were sent to the children’s home afterwards. Mothers’ height (cm) and weight (kg) were provided by the mothers in the questionnaire, which were completed in the same semester.

### Statistical analysis

R i386 3.6.0 for Windows (32/64 bit) was used to conduct all analyses, including both the descriptive analyses and the hierarchical regression analysis. A hierarchical multiple regression analysis was performed to analyze maternal factors, and using children’s factors, including children’s constitution, age, gender and number of children in a family, as the control variables (Table 3). The object (dependent) variable was the children’s BMI, and the explanatory (independent) variables included maternal BMI, age, education level, monthly household income, and marital status. The control variables were first entered (step 1), followed by maternal factors in the second step (step 2). Our interest is to determine the influence caused by maternal factors. Thus, the significant improvement in R^2^ (the proportion of explained variance in children’s BMI by the model). All *p*-values are two-sided. To avoid Type II Error, the alpha level was set at *p* < 0.001. The minimum sample size was calculated by G*power 3.1.9.7. When the predictors are nine, the minimum sample size was 107.
